# Urgent Measures for the Containment of the Coronavirus (Covid-19) Epidemic in the Neurorehabilitation/Rehabilitation Departments in the Phase of Maximum Expansion of the Epidemic

**DOI:** 10.3389/fneur.2020.00423

**Published:** 2020-04-30

**Authors:** Michelangelo Bartolo, Domenico Intiso, Carmelo Lentino, Giorgio Sandrini, Stefano Paolucci, Mauro Zampolini, The Board of the Italian Society of Neurological Rehabilitation (SIRN)

**Affiliations:** ^1^Neurorehabilitation Unit, Department of Rehabilitation, HABILITA Zingonia, Bergamo, Italy; ^2^Unit of Neuro-Rehabilitation, and Rehabilitation Medicine, IRCCS “Casa Sollievo della Sofferenza”, San Giovanni Rotondo, Italy; ^3^Recovery and Functional Reeducation Unit, Rehabilitation Department, Santa Corona Hospital, Savona, Italy; ^4^Neurorehabilitation Unit, IRCCS C. Mondino Foundation, Pavia, Italy; ^5^Department of Brain and Behavioral Sciences, University of Pavia, Pavia, Italy; ^6^IRCCS, Fondazione Santa Lucia, Rome, Italy; ^7^Department of Rehabilitation, USL Umbria 2, Foligno, Italy

**Keywords:** COVID-19, rehabilitation, neurorehabilitation, epidemic, infection, health care, organization

## Abstract

COVID-19 has rapidly become a pandemic emergency, distressing health systems in each affected country. COVID-19 determines the need for healthcare in a large number of people in an extremely short time and, like a tsunami wave, overruns emergency, infectious diseases, and pneumology departments as well as intensive care units, choking healthcare services. Rehabilitation services are also affected by this epidemic which forces radical changes both in the organization and in the operating methods. In the absence of reference literature on this issue, this report aims to provide a background documentation to support physicians and healthcare personnel involved in neurorehabilitation and rehabilitation care.

## Introduction

The spread of coronavirus disease 2019 (COVID-19—COrona VIrus Disease 2019) has become unstoppable and in the last few weeks has reached the epidemiological criteria to be declared a pandemic by the World Health Organization, having widely exceeded 400,000 (updated to March 25 2020 Coronavirus COVID-19 Global Cases by the Center for Systems Science and Engineering at Johns Hopkins University) infected people in the world in over 100 countries (1, 2).

In Italy, in February 2020 the emergence of the COVID-19 epidemic first in Lombardy, and then in the other regions, determined the need to implement containment measures for a phenomenon that in a few days has put a strain on the healthcare system, clogging many emergency, infectious diseases and pneumology departments, as well as intensive care units, with obvious dramatic relapses in the health system's ability to offer adequate assistance to patients with different pathologies (3).

Ever since the coronavirus emergency began in China (4), in the current unavailability of an effective etiological therapy, governments have reacted with the standard measures usually adopted in the event of epidemics, represented by quarantine, and by travel and mobility restrictions for the populations involved in outbreak areas. However, unlike in the past, this is a public health emergency that is developing in a globalized and interconnected world like never before. The infectious disease is developing and spreading in an economic and socio-cultural context characterized by populations that tend to aggregate in highly urbanized and overcrowded contexts and by extreme ease of movement, including intercontinental travel (5, 6). The way in which the COVID-19 epidemic is spreading must also be considered, it has rightly been compared to a real “tsunami” wave (7). In fact, the epidemic is expressed by the tremendous number of infections in an extremely short period of time, thus affecting large portions of the population, which leads to the concentration of a disproportionate number of requests for assistance compared to the emergency response capacity of the health systems.

The epidemic growth that is happening in other European (Spain, France, Germany, etc.) and non-European countries, shows that the challenge is similar for everyone. Nonetheless, the absence of a common pandemic plan in Italy and Europe, with Regions and individual countries that are still adopting different methods of managing the epidemic, runs the concrete risk of dissolving the effectiveness of the stringent measures implemented in different areas or countries. This can also be observed in China, where, in the face of an internal growth of new cases equal to zero over the past few days, new infections have been recorded due to the presence of the so-called “re-entry cases.” It must also be taken into account that the epidemic spread will occur with peaks at different times and areas, within and between the various countries, so the overall consequences will be related to the effectiveness of the different health systems.

Numerous mathematical models for predicting the progress of the epidemic are being proposed in Italy and in the world in an attempt to provide useful tools to decision makers (8). However, beyond the absolute numbers of the infections, the increase in new cases in recent weeks, at least in the most affected Italian regions (Lombardy, Emilia-Romagna, Veneto) and European Countries (Spain and France) seems to show a very similar growth curve, only delayed for a few days compared to each other. However, it is also likely that due to the lack of available, prompt, and reliably diagnostic procedures, the officially identified cases only represent the tip of the iceberg, with a spread of unidentified mild/asymptomatic cases well above the estimates.

The currently still limited number of cases, albeit growing, in the central-southern regions, should teach us not to repeat the error of a *wait-and-see* attitude, but should rather induce us to learn from this “temporal advantage,” promptly implementing all the social distancing measures essential to reduce the circulation of the virus.

One of the most worrying aspects of the COVID-19 epidemic is linked to the involvement of frail and vulnerable people, in particular the elderly, subjects who suffer from multiple comorbidities or chronic diseases and people with disabilities. For this reason, it is essential to address and focus on prevention, health interventions and care in chronically ill patients staying in health care facilities, as well as patients suffering recent functional limitation requiring rehabilitation (or admitted to rehabilitation facilities).

In fact, in an extremely short time compared to the spread of the epidemic, Rehabilitation Facilities need to enact prompt remodellling of the health organization both in the internal structure and in the strategy and approach of care delivery, which presents unique peculiarities within healthcare organizations (9, 10).

Furthermore, in some areas due to the lack of beds, rehabilitation facilities are now being occupied with other patients, with consequent difficulty in hospitalizing patients, for example, discharged from stroke units.

Therefore, in the need to reorganize hospital and outpatient rehabilitation activities (11, 12), this document describes the measures adopted by the rehabilitation structures that first faced the fight against COVID-19, hoping for a rapid spread to all those who find themselves involved in this rampant battle.

## Remodeling of Neurorehabilitation/Rehabilitation Activities During COVID-19 Emergency

The following indications are suggested in order to make the reorganization of rehabilitation activities homogeneous, whether they are carried out in hospitalization or outpatient settings or at home, with the main aim of limiting patient flows within the facilities and maintaining staff safety. For some structures, these indications must take into account territorial and network needs, as well as the possible increase in hospital admission in order to favor discharge from acute care facilities.

## “System” Interventions

The definition of dedicated territorial and local pathways for patients from acute care facilities, separating noCOVID or negative COVID patients from suspected positive or positive Covid cases.

## “Structural” Indications

1. Admissions to hospital wards must be limited to only those that are essential, and in any case supervised by personnel equipped with personal protective equipment. All other accesses must be closed.
2. Posting of notices with behavioral rules at the entrances to and within all the departments [see (13)]
3. Posting of hand hygiene recommendations near hand-sanitizing gel dispensers [see (13)]
4. Staff of external companies are required to comply rigorously and systematically with standard precautions in addition to those provided by air, by droplets, and by contact, as indicated in the behavioral rules.
5. Preparation of extraordinary plans for daily cleaning and sanitization of the rooms.
6. Preparation of plans for extraordinary sanitization and cleaning in cases of access or identification of a “suspected case.”
7. Reorganization of work shifts (medical and non-medical staff) with reduction of activities in order to reduce contact and movements. Where possible, encourage staff to work from home (e.g.,: administrative activities, social worker, etc.). Obligation for everyone to report any symptoms that have arisen recently; in this case, respect home isolation.
8. Strengthening of patient and caregiver support networks, also through information technologies.
9. Emphasis on the role of remote assistance and/or tele-rehabilitation in particular as remote home monitoring for patients who are unable to access rehabilitation hospitals or must be discharged in advance as well as for consulting activities in hospitals or in case of consultation for out-patients
10. For all, monitoring of body temperature <37.5°C—(if higher, do not allow access and indicate home isolation)
11. Even in the absence of fever, it is necessary to subject people to careful triage by explicitly asking for their place of origin and detecting potential contacts.

## Indications for the Activities of Neurorehabilitation/Rehabilitation Units

1. Suspension of caregiver visits to hospitalized patients (underwear, clothes, and necessities will be delivered to hospital staff at the entrance of the building, thus avoiding the need to enter). Exceptional cases must be authorized by clinicians according to the rules of the health management; access in derogation will be managed by the staff in order to avoid any contact and for a limited time.
2. Rehabilitation units will have to keep the doors closed in order to control and direct the flow (if possible all the entrances should be recorded, indicating the time). for all health personnel and visitors with permission, monitoring of body temperature <37.5°C (if higher, do not allow access and give indication of home isolation)
3. For discharges (see rules for access in derogation and compliance with point 2)
4. Suspension of all meeting activities, replaced by the use of telephone or email contact.
5. Clinical interviews with family members by phone or email only.
6. Remodeling of rehabilitation programs, identifying the most relevant goals, among the short-term achievable ones
7. Suspension of all rehabilitation activities that require internal flow (movement between floors or to reach gyms)
8. Carrying out of rehabilitation activities in the patients' room where possible; in the case of gym activities, strictly keep the distance of at least 2 m between the patients.
9. Reduction of the rehabilitation team's activities (keep only those which are strictly necessary, which will be carried out by the clinician together with the coordinator, after collecting updates from the other staff)
10. Education and empowerment of all healthcare professionals involved in rehabilitation team, by means of specific targeted training (e.g., about the correct use of PPE)
11. For all healthcare professionals, it is recommended to “enhance hand hygiene by following the WHO instructions, before and after each patient and whenever the hands move from the patient to another surface” (14)
12. Remember that “Masks with greater protection (FFP2, FFP3) are indicated only in suspicious or full-blown cases, therefore it is suggested not to abuse these devices, so as not to reduce their availability for cases of real need”
13. In the management of suspicious cases (cases with not deferrable treatment) (15), remember.
  - patient with respiratory symptoms (no COVID19): the patient is recommended to wear the surgical mask; keep a distance of at least 1 m or wear a surgical mask;
  - patients suspected or affected by Covid-19: the use of FFP2 or FFP3 mask, protective gown, gloves, eye protection (goggles or face shield) is recommended;
  - patients suspected or affected by Covid-19 during the execution of procedures capable of generating aerosols: the use of FFP3 mask, protective gown, gloves, eye protection (goggles or face shield) is recommended
14. Even in the absence of fever, people must be subjected to careful triage by explicitly asking where they came from and detecting potential contacts.

**NOTE**: Remember that it is possible to carry out “…*respiratory physiotherapy in hospitalization and re-education settings in recent outcomes of surgery, in trauma with fractures and the immediately post-acute phase of cardiac and neurological disabling pathologies (heart attack, stroke, etc.) (with appropriate personal protective equipment due to the impossibility of maintaining a distance of* <*1.5 meters)…*,” for this specific area, please refer to the joint document of the Association of Rehabilitators of Respiratory Insufficiency and the Italian Association of Physiotherapists (AIR and AIFI, Indications for respiratory system physiotherapy in patients with COVID-19 infection) (update of 16/03/2020) (16).

## Indications for Patients Suffering From Recent Onset of Neurological Disorders Requiring Rehabilitation

1. Neuro-rehabilitation/neurological Units located in multi-disciplinary Hospitals can admit patients with sub- acute neurological impairments due to severe acquired brain lesions or stroke coming from an acute unit of the same hospital (Intensive care Units, neuro-surgery, neurology), if they are not affected or suspected by Covid-19.
2. Patients suspected or affected by Covid-19 with neurological impairments requiring rehabilitation and according to clinical conditions (hemodynamic parameters, breathing capacity, consciousness) should be treated in the room (*with appropriate personal protective equipment due to the impossibility of maintaining a distance of* <*1.5 m*)
3. Patients with neurological disorders requiring rehabilitation and coming from acute units outside Hospitals should be admitted if throat and nasal swab resulted negative and after proper time assessment (14 day) without fever and cough suggestive of Covid-19 infection.
4. Neurorehabilitation/rehabilitation Unit located outside general and multi-disciplinary Hospitals should only admit patients with sub-acute neurological disorders negative for Covid-19 infections in order to facilitate prompt availability of intensive care unit.

## Indications for Outpatient and Home Rehabilitation Activities

1. Suspension of all outpatient and/or Day-hospital rehabilitation activities and/or with access from the outside (only activities that cannot be postponed according to clinical judgment are maintained, subject to communication, and approval by the Health Department)
2. Suspension of all activities including those of a freelance type (re-scheduling of visits and activities)
3. Suspension of all home rehabilitation activities except those that cannot be postponed according to clinical judgment.

## Conclusions

The greatest difficulty in applying these indications in the field of neurorehabilitation/rehabilitation is related to the need to find the right balance between the provision of services useful to the patient (in case of not deferrable treatment) and the reduction of the risk of spreading the virus. In this phase of maximum spreading speed, priority must be given to reducing the risk of spreading the infection.

The carrying out of rehabilitation activities in hospital stays, and in services in general, can only be continued in compliance with the needs of the patients and the protection of the health of all staff, as activities require close contact with the patient, or with the production of aerosols and secretions as for respiratory rehabilitation interventions (Joint document AIFI - Commission of Physiotherapists Register, 2020) (17). Finally, please note that the WHO protocols indicate minimum standards for the protection of health workers and each Ministry of Health, based on its own risk assessment, can raise the levels of protection of its personnel.

It must be reiterated that given the specificity of Rehabilitation/Rehabilitative interventions, which necessarily have to take into account the territorial contexts, the above indications must be adapted to the specific realities, while adhering as closely as possible to the recommendations.

In fact, it seems useful to reiterate that the indications and measures to be taken are valid for all and should be applied in all countries and in the different areas as simultaneously as possible, because a non-uniform alignment would also lead to the risk of an inevitable misalignment in the desirable economic restart, with heavy long-term relapses.

## Data Availability Statement

The original contributions presented in the study are included in the article/supplementary materials, further inquiries can be directed to the corresponding author/s.

## Author Contributions

MB conceived the study. MB, DI, CL, GS, SP, and MZ also on behalf of the Authors listed in the Appendix reviewed and discussed the study and approved the last version of the paper.

## Conflict of Interest

The authors declare that the research was conducted in the absence of any commercial or financial relationships that could be construed as a potential conflict of interest.

## Appendix

### Board of Italian Society of Neurorehabiliation (SIRN) in: Board of Italian Society of Neurological Rehabilitation (SIRN)

- Alfonsi E, Clinical Neurophysiology Unit, IRCCS Mondino Foundation, Pavia, Italy.
- Antonucci G, Department of Psychology, Sapienza University of Rome, Via dei Marsi 78, 00185 Rome, Italy; Neuropsychology Center, IRCCS Santa Lucia Foundation, Via Ardeatina 306/354, 00142 Rome, Italy.
- Baricich A, Physical and Rehabilitative Medicine, Department of Health Sciences, University of Eastern Piedmont “A. Avogadro”, Novara, Italy; Physical Medicine and Rehabilitation Unit, University Hospital “Maggiore della Carità”, Novara, Italy
- Casu G, ATS Sardegna, Ospedale San Francesco, Nuoro.
- Ciancarelli I, Department of Life, Health and Environmental Sciences, University of L'Aquila, Nova Salus S.r.l., L'Aquila, Italy.
- Cisari C, Physical and Rehabilitative Medicine, Department of Health Sciences, University of Eastern Piedmont “A. Avogadro”, Novara, Italy; Physical Medicine and Rehabilitation Unit, University Hospital “Maggiore della Carità,” Novara, Italy
- De Tanti A, Centro Cardinali Ferrari, Santo Stefano Riabilitazione, Fontanellato, Italy.
- Estraneo A, Neurology Unit, Santa Maria della Pietà General Hospital, Nola, Italy.
- Ferraro F, section of Neuromotor Rehabilitation, Department of Neuroscience, ASST Carlo Poma, Mantova, Italy.
- Gandolfi M, Neurorehabilitation Unit, Department of Neurosciences, University Hospital of Verona, Verona, Italy; Neuromotor and Cognitive Rehabilitation Research Center, Physical and Rehabilitation Medicine section, Department of Neurosciences, Biomedicine and Movement Sciences, University of Verona, Verona, Italy.
- Invernizzi M, Physical and Rehabilitative Medicine, Department of Health Sciences, University of Eastern Piedmont “A. Avogadro,” Novara, Italy
- Iolascon G, Physical and Rehabilitation Medicine Department, Napoli University, Naples, Italy.
- Martinuzzi A, Acquired Neuropsychological Disease Rehabilitation Unit, Scientific Institute, IRCCS Eugenio Medea, Pieve di Soligo, Italy; Severe Developmental Disabilities Unit, Scientific Institute, IRCCS Eugenio Medea, Conegliano, Italy.
- Morelli D, IRCCS Santa Lucia Foundation, Rome, Italy.
- Morone G, IRCCS Santa Lucia Foundation, Rome, Italy.
- Munari D, Neurorehabilitation Unit, Department of Neurosciences, University Hospital of Verona, Verona, Italy.
- Nordio S, IRCCS San Camillo Hospital, Venice, Italy.
- Picelli A, Neuromotor and Cognitive Rehabilitation Research Center, Physical and Rehabilitation Medicine section, Department of Neurosciences, Biomedicine and Movement Sciences, University of Verona, Verona, Italy; Neurorehabilitation Unit, Department of Neurosciences, University Hospital of Verona, Verona, Italy
- Scarponi F, Department of Rehabilitation, San Giovanni Battista Hospital, Foligno, Perugia, Italy
- Pingue V, Neurorehabilitation and Spinal Unit, Institute of Pavia, Istituti Clinici Scientifici Maugeri IRCCS, Pavia, Italy
- Solaro C, Department of Rehabilitation, Mons L Novarese Hospital, Moncrivello, Italy; Department of Head and Neck, ASL 3 Genovese, Genoa, Italy.
- Trompetto C, Department of Neuroscience, Rehabilitation, Ophthalmology, Genetics, Maternal and Child Health, University of Genoa, Italy; Department of Neuroscience, IRCCS Ospedale Policlinico San Martino, Genoa, Italy.
